# Inhibiting PSMα-induced neutrophil necroptosis protects mice with MRSA pneumonia by blocking the *agr* system

**DOI:** 10.1038/s41419-018-0398-z

**Published:** 2018-03-02

**Authors:** Ying Zhou, Chao Niu, Bo Ma, Xiaoyan Xue, Zhi Li, Zhou Chen, Fen Li, Shan Zhou, Xiaoxing Luo, Zheng Hou

**Affiliations:** 1 Department of Pharmacology, School of Pharmacy, Fourth Military Medical University, Xi’an, China; 2 Department of Pharmacology, Xi’an Medical University, Xi’an, China; 3 Henan Eye Institute, Henan Provincial People’s Hospital, Zhengzhou, China; 4 Department of Clinical Laboratory Medicine, Xijing Hospital, Fourth Military Medical University, Xi’an, China

## Abstract

Given its high resistance, enhanced virulence, and high transmissibility, community-associated methicillin-resistant *Staphylococcus aureus* (CA-MRSA) pneumonia is highly associated with high morbidity and mortality. Anti-virulence therapy is a promising strategy that bypasses the evolutionary pressure on the bacterium to develop resistance. RNAIII-inhibiting peptide (RIP), as an accessory gene regulator (*agr*)-specific inhibitor, significantly restricts the virulence of *S. aureus* and protects infected mice from death by blocking the *agr* quorum sensing system. The protective effects of RIP on the neutropenic mice completely disappeared in a neutrophil-deleted mouse infection model, but not in the macrophage-deleted mice. This result confirmed that the in vivo antibacterial activity of RIP is highly associated with neutrophil function. Phenol-soluble modulins (PSMs), as major leukocyte lysis toxins of CA-MRSA, are directly regulated by the *agr* system. In this experiment, PSMα1, 2, and 3 significantly induced neutrophil necroptosis by activating mixed lineage kinase-like protein (MLKL) phosphorylation and increasing lactate dehydrogenase release. The *S. aureus* supernatants harvested from the *agr* or *psmα* mutant strains both decreased the phosphorylation level of MLKL and cell lysis. PSMα1-mediated neutrophil lysis was significantly inhibited by necrosulfonamide, necrostatin-1, TNFα antibody, and WRW4. These results showed PSMα1 induced necroptosis depends on formylpeptide receptor 2 (FPR2)-mediated autocrine TNFα. Moreover, the neutrophil necroptosis induced by *S. aureus* was significantly suppressed and pneumonia was effectively prevented by the blockage of *agrA* and *psmα* expression levels. These findings indicate that PSMα-induced necroptosis is a major cause of lung pathology in *S. aureus* pneumonia and suggest that interfering with the *agr* quorum sensing signaling pathway is a potential therapeutic strategy.

## Introduction

*Staphylococcus aureus* (*S. aureus*) is an important bacterial pathogen that causes various respiratory tract infections in both adult and pediatric populations^1^. To date, methicillin-resistant *S. aureus* (MRSA) has instigated an antibiotic resistance crisis to commonly used antibiotics in the clinics due to overuse. Linezolid and vancomycin are the most reliable therapeutic agents against MRSA pneumonia^2, 3^; however, vancomycin-resistant *S. aureus* (VRSA) and linezolid-resistant *S. aureus* (LRSA) have alarmingly emerged in severe MRSA pneumonia cases^4, 5^. Moreover, the development of antibiotic resistance is associated with high morbidity and mortality risk, particularly in the intensive care unit^6^. Therefore, novel strategies are urgently needed for treating and preventing invasive MRSA infections.

Quorum sensing is a bacterial intercellular communication mechanism that controls the pathogenesis of many organisms by regulating gene expression. This mechanism targets many virulence strategies and components, such as virulence determinants, biofilm formation, and drug resistance^7^. The quorum sensing system has become an attractive target for the development of novel anti-infective agents and helps reduce the potential development of bacterial resistance^8, 9^. In staphylococci, the heptapeptide RNAIII-inhibiting peptide (RIP) can inhibit the activation of the accessory gene regulator (*agr*) quorum sensing system and decrease staphylococcal virulence by interfering with the phosphorylation of TRAP (target of RNAIII-activating peptide)^8, 10^.

Phenol-soluble modulins (PSMs) are recently discovered amphipathic alpha-helical peptides that are mainly expressed by highly virulent *S. aureus* and are closely related to staphylococcal pathogenesis. The enhancement of PSMs production is strictly and directly promoted by the *agr* quorum sensing regulation system at high cell densities^11^. PSMs are produced at considerable amounts in community-associated MRSA (CA-MRSA), such as LAC (USA300), but are expressed at lower amounts in hospital-associated MRSA (HA-MRSA)^12, 13^. HA-MRSA with overexpressed α-type PSMs causes neutrophil lysis activity similar to that caused by CA-MRSA strains^14, 15^. Therefore, α-type PSMs are mainly responsible for the pronounced in vivo leukocidal activity in addition to chemotactic and proinflammatory activities^12, 13^. Moreover, α-type PSMs have been demonstrated to dramatically influence the infectivity of CA-MRSA in mouse bacteremia, skin abscess, and peritonitis models^13, 14, 16^.

Neutrophil, as an important host innate immune cell, plays a key role in staphylococcal infections by phagocytosis. However, 15–50% of the initial ingested *S. aureus* survives within the neutrophil phagosome and likely contributes to disease pathogenesis. In 2014, Greenlee-Wacker et al. first reported on the intraphagosomal *S. aureus* that induces programmed necrosis in neutrophils^17^. This programmed necrosis further exacerbates staphylococcal disease by releasing viable *S. aureus* and neutrophil constituents^17^. Viable *S. aureus* can propagate infection to distant sites, and neutrophil constituents may cause substantial tissue damage^18, 19^.

Based on the evidence from these studies, we hypothesized that the *agr* blocking strategy may inhibit neutrophil necroptosis by reducing the critically virulent PSMα release and promoting *S. aureus* clearance. So we investigated the possible anti-infective mechanisms of RIP as an *agr* system inhibitor in this experiment. Data showed that PSMα-induced neutrophil necroptosis was blocked, LAC elimination was enhanced in the pneumonia mouse model, tissue damage was alleviated, and the mouse survival rate was greatly improved by interfering with the *agr* quorum sensing system.

## Results

### Bacterial growth was not inhibited by RIP in vitro

Six *Staphylococcus* strains were cultured in Mueller-Hinton (MH) Broth medium in the absence or presence of RIP at different concentrations. MIC assay results showed that RIP did not exert bactericidal effects at 256 μg/mL in vitro (Table 1). The growth curve demonstrated that RIP did not inhibit the growths of the six strains in vitro at concentrations up to 1000 μg/mL. Meanwhile, vancomycin was used as positive control, which completely inhibited the growths of the six tested strains at 64 μg/mL (Supplementary Figure 1).

**Table 1:** MICs of RIP and five antibiotics on six *Staphylococcus* strains in MH broth culture

Strains	MIC (μg/mL)					
	RIP	VAN	OXA	CIP	CAZ	LVX
*S. aureus* ATCC29213	>256	0.5	0.25	≤0.25	8	≤0.25
LAC (USA300)	>256	0.5	2	8	256	4
Mu50	>256	8	16	32	256	8
MRSA XJ75302	>256	0.5	>256	16	256	8
*S. epidermidis* ATCC14990	>256	0.5	0.125	0.125	16	0.125
MRSE XJ75284	>256	1	>256	8	256	8

*VAN* vancomycin, *OXA* oxacillin, *CIP* ciprofloxacin, *CAZ* ceftazidine, *LVX* levofloxacin

### MRSA-infected mice were protected by blocking the *agr* system

**Fig. 1:**
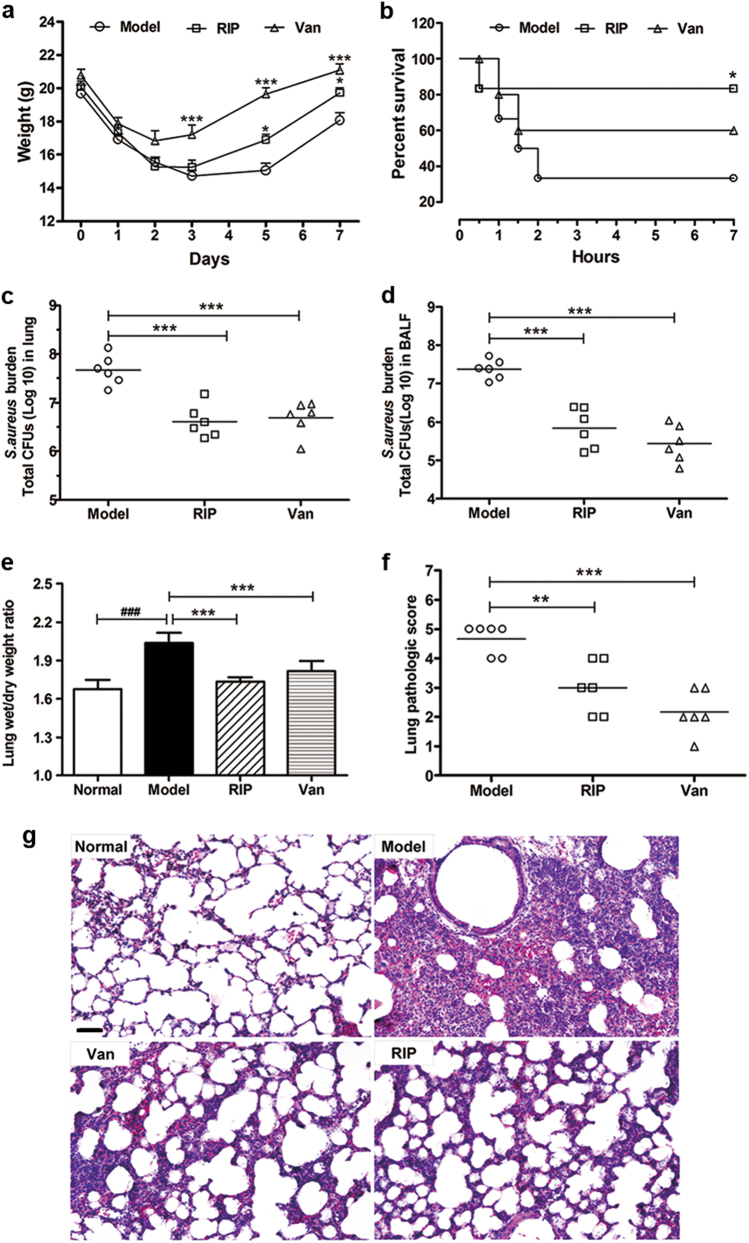
LAC-infected pneumonia mice were protected by blocking the *agr* system. **a** The body weight of LAC-infected mice recovered after treated by 20 mg/kg RIP or 10 mg/kg vancomycin (*n* = 7). **b** Survival of BALB/c mice nasally inoculated with LAC (3 × 10^7^ CFU) and treated with PBS, RIP, or vancomycin by i.p. injection at 1 and 6 h after infection (*n* = 12). **c**, **d** The number of CFU in the lung (**c**) or in the BALF (**d**) was calculated from the number of colonies growing on plates (*n* = 6). **e** Effects of RIP on lung injury in LAC-infected mice were analyzed by Lung wet/dry weight (*n* = 6). **f** After 24 h infection, the left lung of infected mice were harvested, fixed, stained with HE, and analyzed microscopically. The total pathologic score for each mouse was calculated. Severity was graded on a scale of 0–5, with 0 representing normal lung and 5 representing severe pneumonia (*n* = 6). **g** Lung histopathologic examination of LAC-induced pneumonia 24 h after bacterial challenge in mice. The original magnification is ×200. The bar length represents 50 μm. Data were shown as mean (**c**, **d**, **f**), mean ± SD (**e**), or mean ± SE (**a**). *P < 0.05, **P < 0.01, ***P < 0.0001 vs. model group; ^###^*P* < 0.0001 vs. normal group

RIP significantly accelerated the body weight recovery of the LAC-infected mice relative to that of the model group (Fig. 1a). Administering 20 mg/kg RIP to the LAC-infected group significantly improved the animal survival rate from 33.3% for the control groups to 83.3% (Fig. 1b). Survival was associated with reduced bacterial titers in the lung and bronchoalveolar lavage fluid (BALF) of the infected mice (Fig. 1c, d). To assess the pathological changes, we performed hematoxylin–eosin (HE) staining, measured the lung wet/dry weights, and applied a lung injury score system in our study. Results showed that RIP can significantly reduce pulmonary edema and improve the lung injury score (Fig. 1e–g).

### *agrA*, *psmα*, and *psmβ* expression levels were inhibited by blocking the *agr* system

**Fig. 2:**
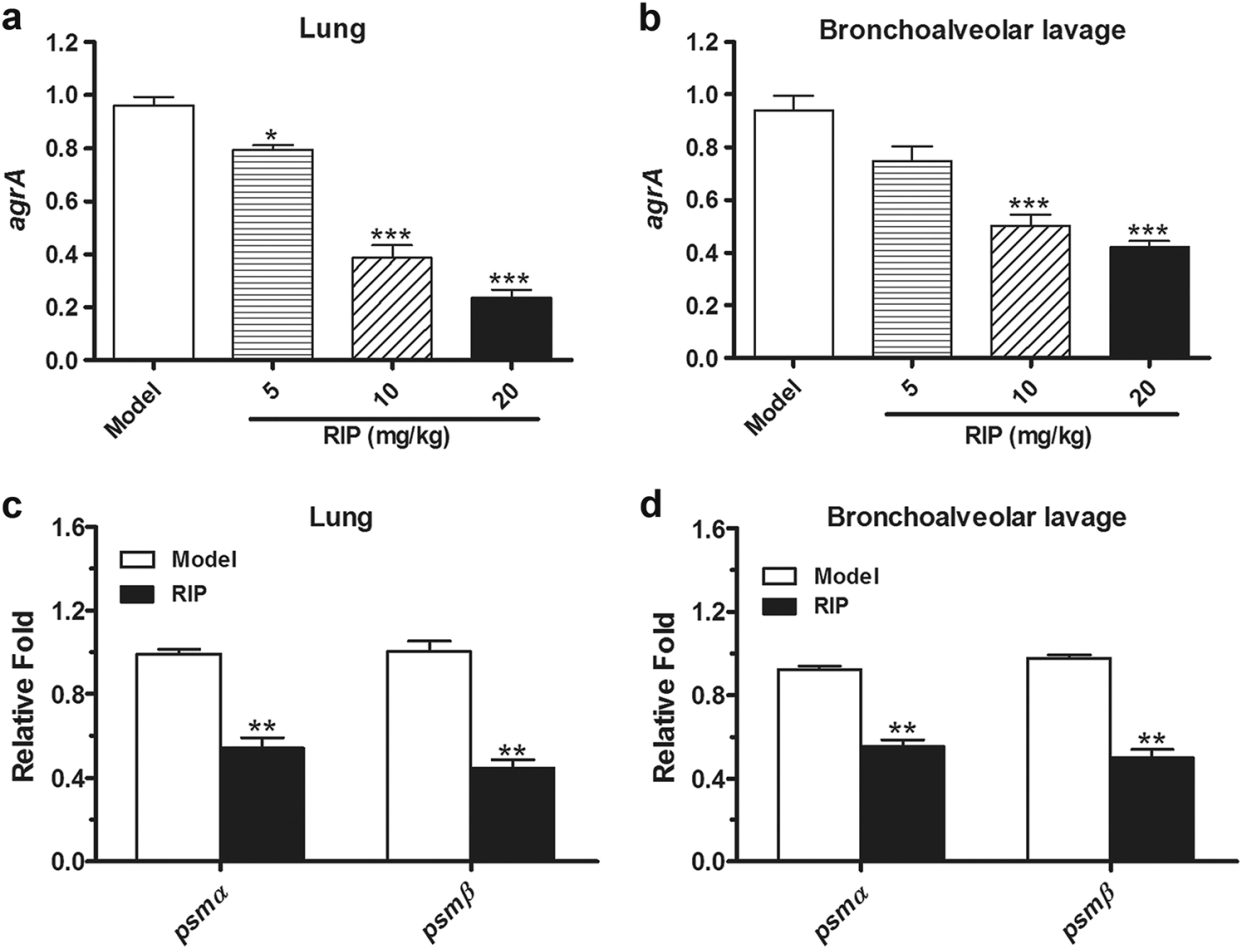
The quantitative RT-PCR analysis of the *agrA, psmα*, and *psmβ* genes in LAC isolated from infected mice. **a**, **b** The mRNA expression levels of *agrA* in LAC that isolated from lung tissue or bronchoalveolar lavage fluid in LAC-infected mice following treatment with or without RIP. **c**, **d** The mRNA expression levels of *psmα* and *psmβ* in LAC that isolated from lung tissue or bronchoalveolar lavage fluid. The mRNA levels were quantified after comparison with an internal gene (*16S rRNA*). Data are expressed as the mean ± SE (*n = *3). *P < 0.05, **P < 0.01, ***P < 0.0001 vs. model group

Virulence factors of PSMs are regulated by *agrA* in *S. aureus*. RIP reduced the expression of *agrA* dose-dependently (Fig. 2a, b). Moreover, expression levels of *agrA*-dependent virulence factors *psmα* and *psmβ* were decreased (Fig. 2c, d).

### Neutrophil is essential to RIP’s in vivo protective effect

**Fig. 3:**
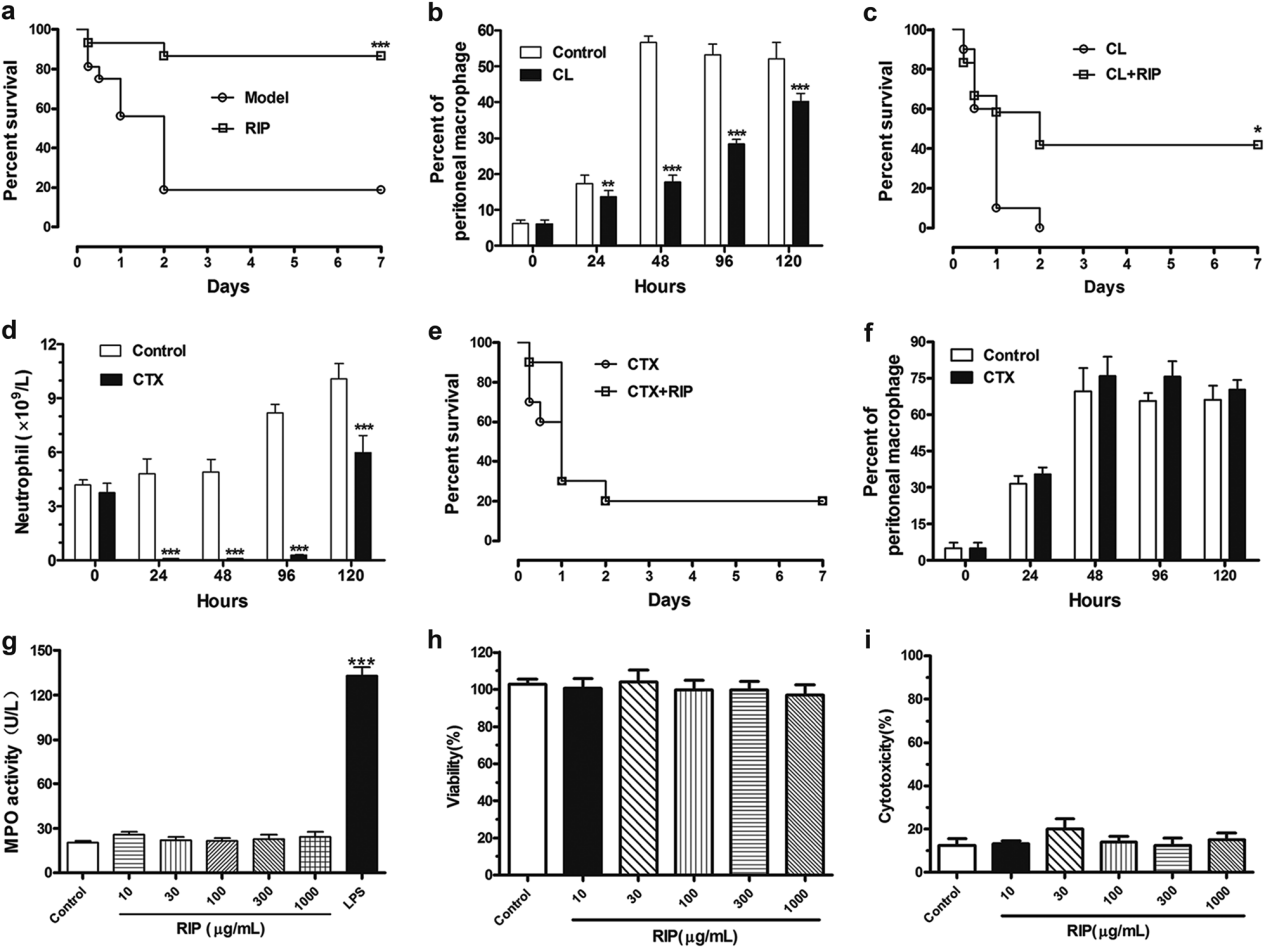
Neutrophil is essential to RIP’s in vivo protective effect. **a** Survival of LAC-infected mice treated with 20 mg/kg RIP (*n* = 15). The model group was given the same volume of PBS (*n* = 16). **b**, **f** The proportion of macrophages in the peritoneal lavage fluid of mice. BALB/c mice were injected intravenously with 150 μL clodronate liposomes (CL) and 200 mg/kg cyclophosphamide (CTX), and subsequently challenged with 3 × 10^7^ CFU LAC. The proportion of macrophages was analyzed by flow cytometry (*n* = 3). **c** Survival rate of the macrophage-deleted mice infected with LAC and then treated by 20 mg/kg of RIP at 1 and 6 h after infection (*n* = 10). **d** Neutrophil depletion was performed in BALB/c mice via administration of 200 mg/kg CTX, and then the mice were challenged by 3 × 10^7^ CFU LAC. The number of neutrophil in the blood of mice was analyzed with an Abbott Cell-Dyn 3700 system (*n* = 3). **e** Survival rate of the neutrophils-deleted mice infected with LAC and then treated with 20 mg/kg RIP at 1 and 6 h after infection (*n* = 10). **g** Neutrophils were incubated with 10, 30, 100, 300, and 1000 μg/mL RIP or 50 ng/mL LPS alone for 30 min at 37 °C. MPO enzyme activity was measured spectrophotometrically in the cell-free supernatants (*n* = 4). **h**, **i** After human neutrophils were pretreated with various concentrations of RIP for 24 h at 37 °C and 5% CO_2_, cell proliferation was measured by CCK-8 (*n* = 8) and the release of LDH amount was determined (*n* = 6). Data were shown as mean ± SD (**b**, **d**), or mean ± SE (**f**–**i**). **P < 0.01, ***P < 0.0001 vs. control group

After a single tail-vein injection of 150 μL clodronate liposomes or 200 mg/kg CTX, most neutrophils or macrophages were both depleted in mice at 24, 48, 96, and 120 h (Fig. 3b, d), and CTX had no effect on macrophages in mice peritoneal lavage (Fig. 3f). RIP can significantly improve the survival rate of LAC-infected mice from 18.8% for the model groups to 86.7% (Fig. 3a). RIP also exerted a protective effect after the deletion of macrophages with 41.7% survival rate (Fig. 3c). However, the protective effects of RIP on the neutropenic mice were completely lost, and all the mice died within 2 days in the model and RIP-treated groups (Fig. 3e). Compared with the control group, the 10, 30, 100, 300, and 1000 μg/mL concentrations of RIP did not affect the myeloperoxidase (MPO) activity of neutrophils, whereas LPS significantly increased the MPO activity of the neutrophils (Fig. 3g). Meanwhile, RIP did not affect the neutrophils’ viability and LDH release compared with the control group (Fig. 3h, i). Thus, the RIP did not directly activate the neutrophils.

### LAC supernatant induces neutrophil necroptosis

**Fig. 4:**
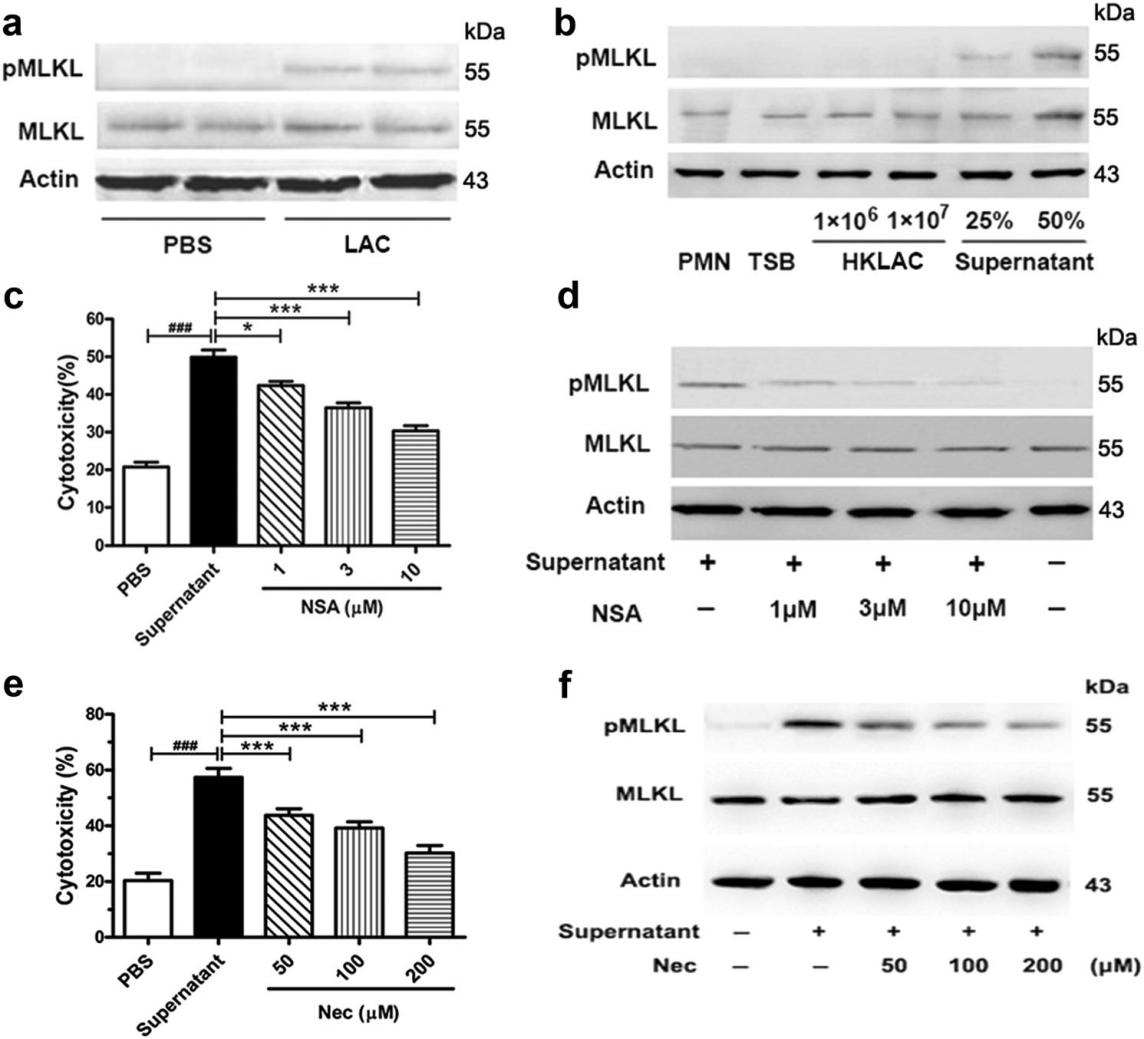
LAC induced necroptosis in neutrophil. **a**, **b** Human neutrophils were stimulated with PBS, LAC (MOI 10), LAC culture supernatant, or HKLAC. Level of phosphorylated MLKL (pMLKL), MLKL, and β-actin in neutrophil lysate were detected by western blot. **c**, **e** Human neutrophils pretreated with 1, 3, and 10 μM necrosulfonamide (NSA) or 50, 100, and 200 μM Nec for 1 h at 37 °C and 5% CO_2_, and then were stimulated with bacterial supernatant for 1 h. The release of LDH by neutrophils was detected (*n* = 3). **d**, **f** Levels of pMLKL, MLKL, and β-actin in neutrophil lysate in (**c**) or (**e**) treatment were detected by western blot. Data were shown as mean ± SE (**c**, **e**). *P < 0.05, ***P < 0.0001 vs. supernatant treated group; ^###^*P* < 0.0001 vs. PBS-treated group

A *S. aureus* and neutrophil co-culture system was utilized to investigate the possible mechanisms underlying the antimicrobial effects of the *agr* inhibitor. The level of phosphorylation-mixed lineage kinase-like protein (pMLKL) was increased in human neutrophils regardless of the LAC or LAC supernatant co-incubation of the neutrophils (Fig. 4a, b). Moreover, the levels of pMLKL and cell lysis were prevented by the MLKL inhibitor Necrosulfonamide (NSA) or receptor-interacting protein kinase 1 (RIPK1) inhibitor Necrostatin-1 (Nec) in a concentration-dependent manner (Fig. 4c–f). The above-mentioned data indicated that the *S. aureus* culture supernatant may induce neutrophil necroptosis.

### PSMα1 induces neutrophil necroptosis

**Fig. 5:**
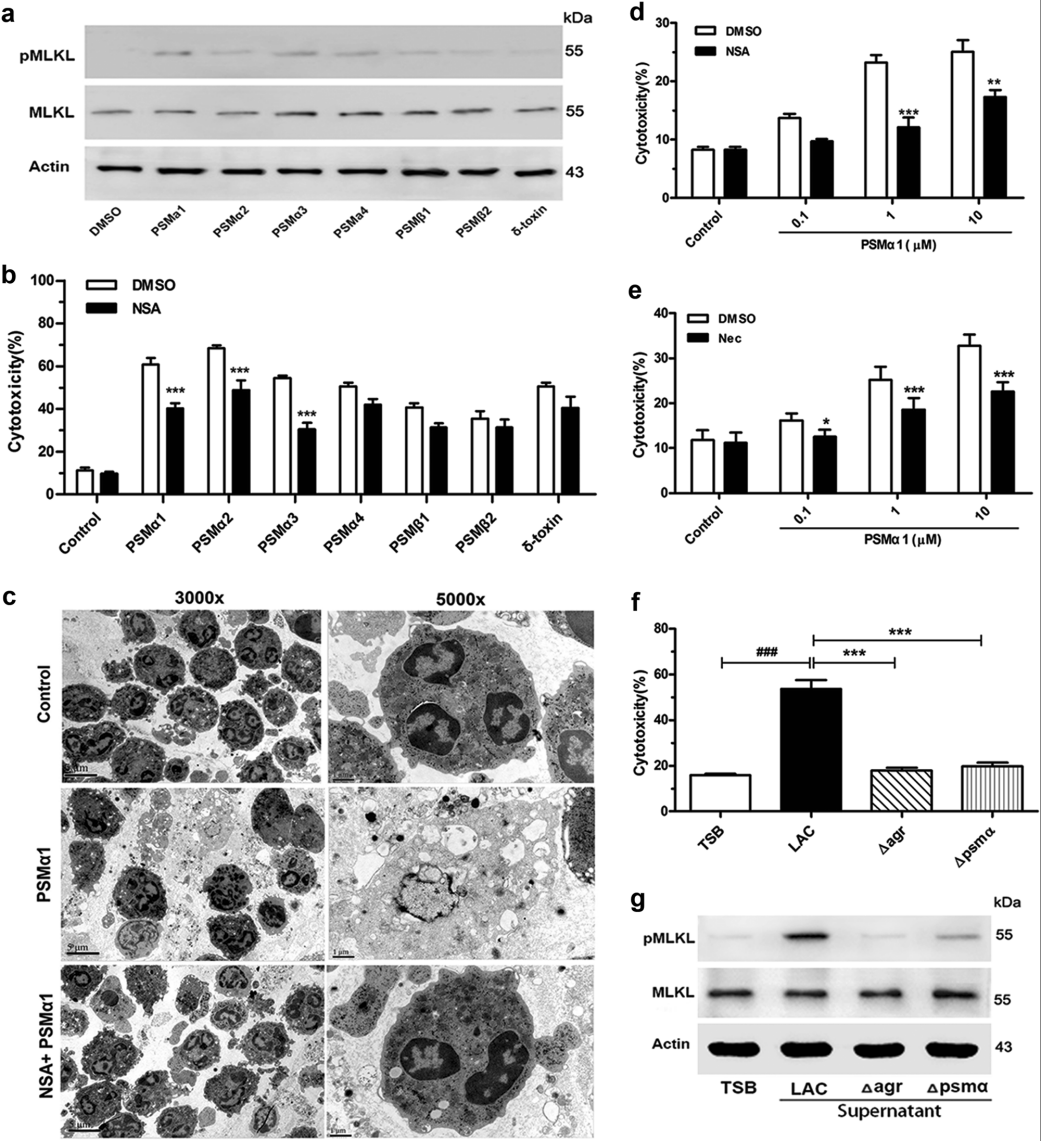
Neutrophil necroptosis was induced by PSMs. **a** Expression levels of pMLKL and MLKL in neutrophils lysis after treated by 1 μM PSMs peptide for 1 h at 37 °C and 5% CO_2_. **b** Neutrophil were respectively stimulated with 1 μM PSMs peptide for 1 h at 37 °C and 5% CO_2_, with or without 10 μM NSA pretreatment for 1 h. The release of LDH by neutrophil was detected (*n* = 6). **c** Neutrophils examination by a TEM after treating with 1 μM PSMα1 with or without 10 μM NSA. The original magnification is ×3000 or ×5000. **d**, **e** Neutrophil cells were respectively stimulated with 0.1, 1, and 10 μM PSMα1, with or without 10 μM NSA or 200 μM Nec pretreatment for 1 h. The release of LDH by neutrophil was detected (*n* = 6). **f**, **g** Human neutrophils were stimulated with LAC or its mutant strains (*∆psmα* or *∆agr*) bacteria supernatant for 1 h at 37 °C and 5% CO_2_. The release of LDH was detected (*n* = 6). Level of pMLKL, MLKL, and β-actin in neutrophil were detected by western blot. Data were shown as mean ± SE (**b**, **d**– **f**). *P < 0.05, **P < 0.01, ***P < 0.0001 vs. control group; ^###^*P <* 0.0001 vs. TSB-treated group

To investigate the roles of these PSMs in inducing neutrophil necroptosis, we incubated these seven synthetic peptides (Supplementary Table 1) with neutrophils. Results showed that PSMα1, 2, and 3 can increase the level of pMLKL and the release of LDH in neutrophils, which can be significantly inhibited by NSA (Fig. 5a, b). The morphology of neutrophils was observed under transmission electron microscopy (TEM). After treatment with PSMα1, the cell volume increased, the surface morphology became irregular, and the organelles and cell submicrons disappeared (Fig. 5c). However, the morphology and structure of the neutrophils resembled those of the control neutrophil cells after NSA pretreatment (Fig. 5c). Cytotoxicity results showed that MLKL inhibitor NSA or RIPK1 inhibitor Nec, significantly reversed the neutrophil lysis caused by PSMα1 (Fig. 5d, e). Moreover, culture supernatants harvested from the *agr* or *psmα* mutant decreased the release of LDH and the expression level of pMLKL in neutrophils compared with LAC group (Fig. 5f, g).

### PSMα1-induced neutrophil necroptosis was blocked by WRW4 or anti-TNFα

**Fig. 6:**
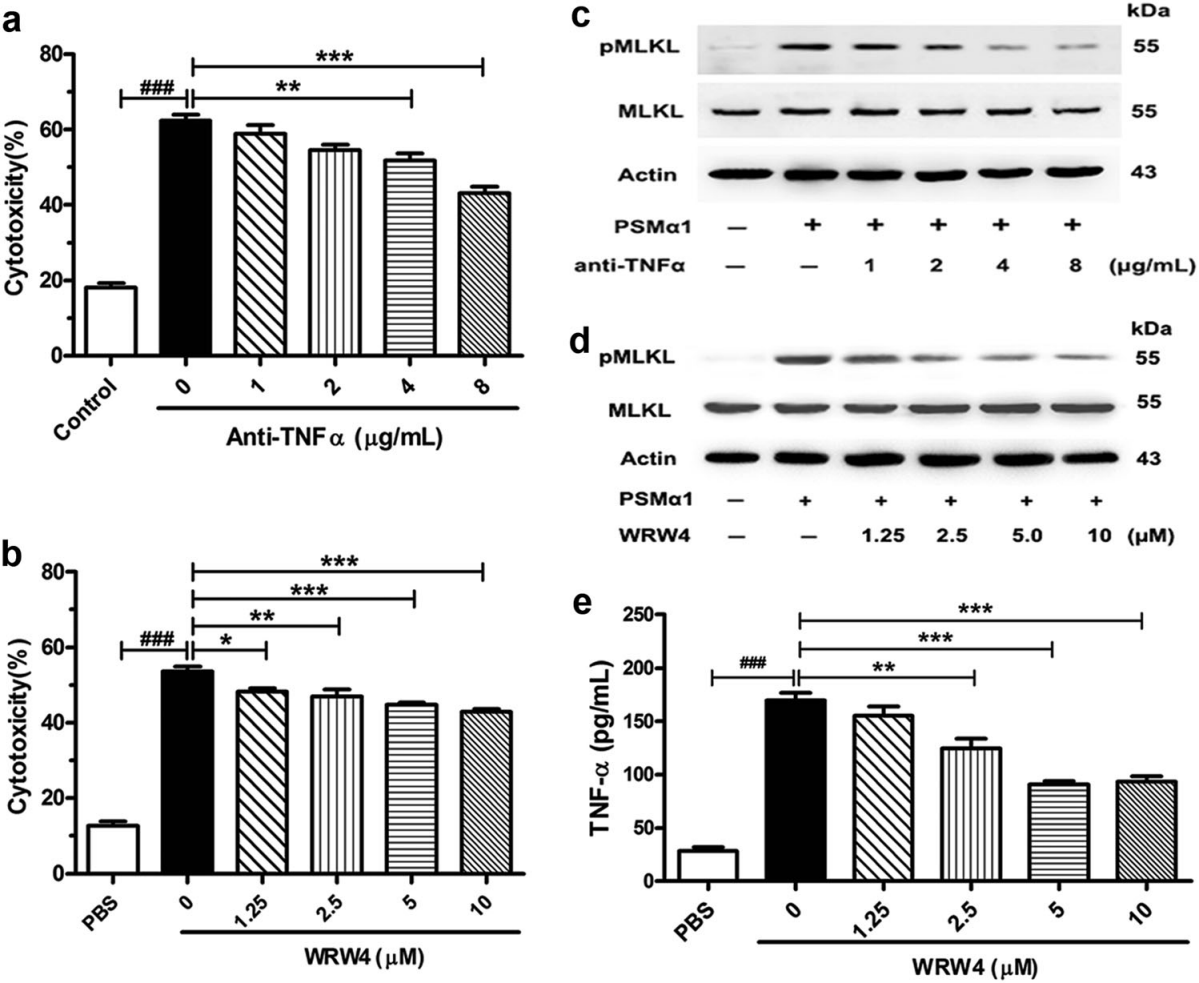
PSMα1-induced neutrophil necroptosis was blocked by WRW4 or anti-TNFα. **a, c** Neutrophil were treated with PBS or TNFα neutralizing antibody at indicated concentrations for 1 h prior to the incubation with 1 μM PSMα1 for 1 h. The release of LDH was detected (*n* = 6) and the level of pMLKL, MLKL and β-actin in neutrophil were detected by western blot (*n* = 3). **b, d, e** Neutrophil were treated with PBS or WRW4 at indicated concentrations for 1 h prior to the incubation with 1 μM PSMα1 for 1 h. The release of LDH was detected (*n* = 6), the level of pMLKL, MLKL and β-actin in neutrophil were detected by western blot (*n* = 3), and the level of TNFα was detected by ELISA assay (*n* = 8). Data were shown as mean ± SE (a, b, e). *P < 0.05, **P < 0.01, ***P < 0.001 vs. control group; ^###^*P* < 0.0001 vs. PBS-treated group

To figure out whether or not TNFα participates in PSMα1-induced necroptosis in neutrophil cells, the cells were pretreated with TNFα antibody (anti-TNFα) and subsequently stimulated with PSMα1. Our data showed that addition of an increasing amount neutralizing anti-TNFα to the culture medium inhibited obviously PSMα1-induced LDH release and MLKL phosphorylation (Fig. 6a, c). Consistently, blocking formylpeptide receptor 2 (FPR2) by WRW4 also markedly prevented PSMα1-induced neutrophil necroptosis and decreased the level of TNFα in cell culture (Fig. 6b, d, e) in a concentration-dependent manner.

### Inhibiting necroptotic cell death protects the infected mice

**Fig. 7:**
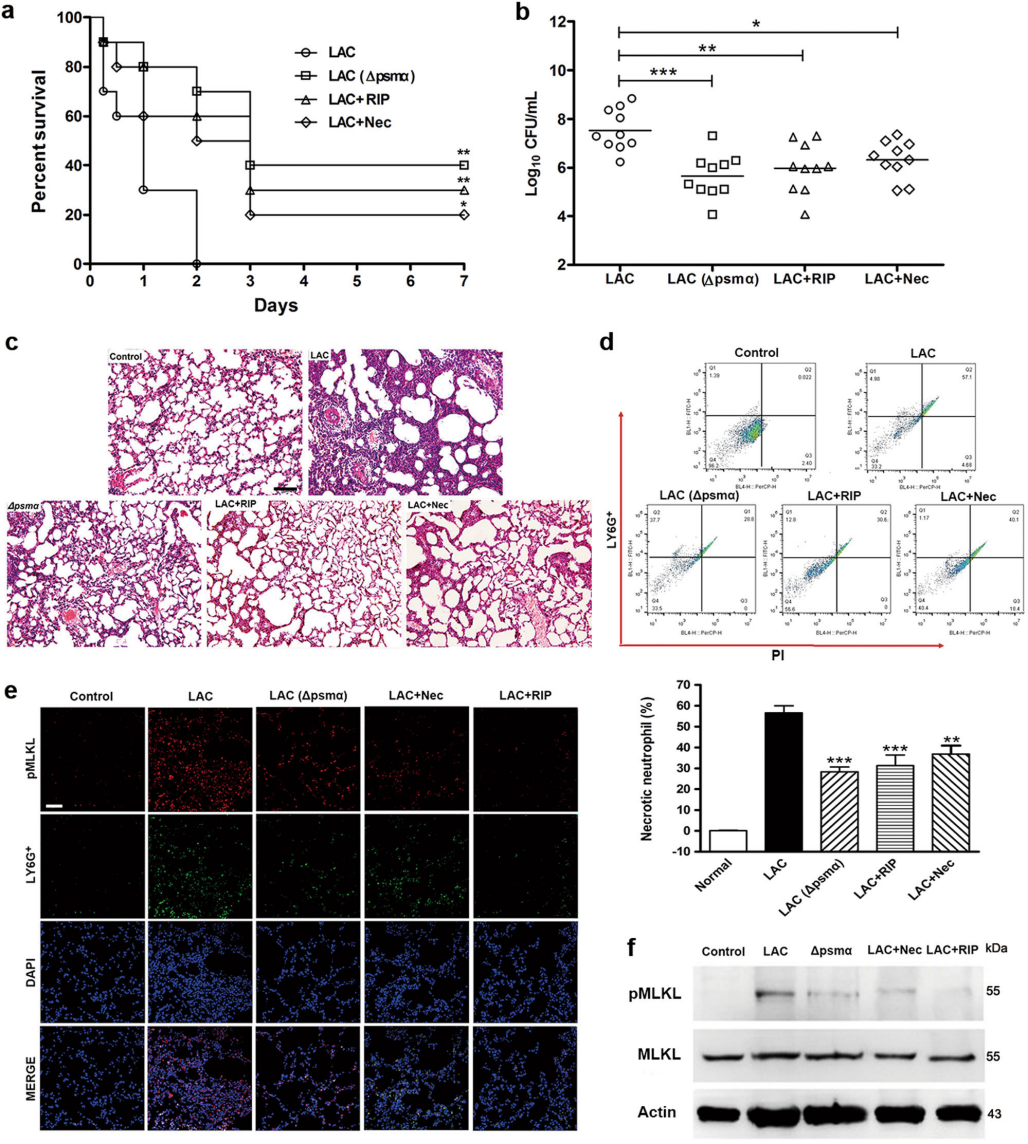
Inhibiting necroptotic cell death protects the infected mice. BALB/c mice nasally infected by 5 × 10^7^ CFU LAC were treated by 20 mg/kg RIP or 15 mg/kg Nec at 1 and 6 h after infection. Or BALB/c mice infected by 5 × 10^7^ CFU *∆psmα*. **a** The survival rate of infected mice (*n* = 10). **b** After infection 24 h the bacterial CFU in the lung cultures were quantified (*n* = 10). **c** After 24 h infection, the lung of mice was harvested, fixed, stained with HE staining, and analyzed microscopically. The original magnification is ×200. The bar length represents 50 μm. **d** Flow cytometry was used to analyze the proportion of necrotic neutrophils in lung tissues of BALB/c mice after infection 24 h (*n* = 6). **e** Immunofluorescence microscopy is used to localize the pMLKL in the lung tissue of BALB/c mice in different groups. The bar length represents 50 μm. **f** Levels of pMLKL, MLKL, and β-actin in neutrophils isolated from bronchoalveolar lavage of BALB/c mice were detected. Data were shown as mean (**b**) or as mean ± SE (**d**). *P < 0.05, **P < 0.01, ***P < 0.0001 vs. LAC-infected group

A pneumonia mouse model was established using wild-type LAC or *Δpsmα*. LAC-infected mice were intraperitoneally injected with 20 mg/kg RIP or 15 mg/kg Nec. LAC-infected mice treated with RIP, Nec, or mice infected by *Δpsmα* strain achieved higher survival rates relative to those of the LAC-infected group (Fig. 7a). The number of *S. aureus* colony-forming units (CFU) in the lung tissue of the LAC-infected mice was significantly higher than that after RIP, Nec, or *Δpsmα* treatment (Fig. 7b). HE data showed that the LAC-infected mice presented with less lung injury after RIP and Nec administration or *Δpsmα* infection than before these treatments were given (Fig. 7c). Flow cytometry data revealed that the percentage of necrotic neutrophils in the lung tissue of the LAC-infected mice increased to 56.6%, but became 28.3%, 31.3%, or 41.9% after *Δpsmα*, RIP, or Nec treatment, respectively (Fig. 7d). Immunofluorescence results showed that the necroptotic neutrophils were less in the lung tissue than in the model mice after the LAC-infected mice were injected with RIP or Nec or infected with *Δpsmα* (Fig. 7e). Western blot analysis revealed that the neutrophils in the LAC-infected mice decreased in pMLKL level after *Δpsmα*, Nec, or RIP treatment (Fig. 7f).

## Discussion

The widespread use of common antimicrobial agents against MRSA has triggered a surge in multidrug-resistant *S. aureus*. The cost of treating MRSA infections is higher than that of methicillin-susceptible *S. aureus* and inflicts tremendous economic and medical burden on patients and the healthcare system. Anti-virulence therapy is a promising strategy that targets *S. aureus* pathogenicity rather than viability. As such, this method controls the severity of an infectious disease while bypassing the evolutionary pressure on the bacterium to develop resistance. RIP, as an *agr* quorum sensing system in staphylococcal strains, inhibits the expression of many virulence factors and has exerted individual or synergistic protective effects in several mouse sepsis models^20, 21^. In the present study, the protective effects of RIP were confirmed in a pneumonia infection model. After RIP treatment in vivo, the bacterial burden was reduced significantly in the lung and BALF of infected mice (Fig. 1). Moreover, the pathological damage to the infected organs was alleviated, and the general weight and survival rate were improved significantly (Fig. 1).

Most PSMs are regulated tightly by the *agr* system. The core peptide sequence of PSMs is highly conserved in *S. aureus* strains. PSMs are categorized as α-type and β-type. The promoters of *psmα* and *psmβ* genes have been confirmed to be directly regulated by AgrA^22^. PSMα is a toxin associated with severe necrotizing pneumonia^23, 24^. PSMα is produced by most staphylococci and released at high levels by CA-MRSA^12, 13^. Studies have shown that HA-MRSA strains that overexpress the *psmα* genes more readily cause infection than that with the regular express of the *psmα* genes^14, 15^. Meanwhile, the β-type PSMs of *Staphylococcus epidermidis* play a key role in biofilm development^25^. All of these genes are tightly regulated by *agr* and are particularly important in the *S. aureus* pathogenesis. Thus, the expression and virulence of these genes were investigated. The *agrA* expression was markedly decreased by RIP in a concentration-dependent manner (Fig. 2). Moreover, *agrA* blockade significantly inhibited the expression of *agrA*-regulated virulence genes, including *psmα* and *psmβ*, in *S. aureus* (Fig. 2).

Neutrophils and macrophages are the two most important cellular defenses against invading *S. aureus*. Their phagocytosis is a highly effective effect for *S. aureus* clearance^26–28^. Moreover, RIP exhibits no antibacterial activity in vitro, thus, a neutrophil- or macrophage-deleted mouse infection model was used to investigate which immune cell plays a critical role in the protective effects of RIP in vivo. Our results revealed that relative to normal LAC-infected mice, the LAC-infected mice treated with RIP exerted protective effects even after their macrophages were deleted (Fig. 3). However, the protective effects of RIP completely disappeared in the neutropenic mice. All these data confirmed that the in vivo antibacterial activity of RIP is highly associated with neutrophil function. To exclude the direct effects of RIP on neutrophils, we detected the MPO activity, cell viability and cytotoxicity of neutrophils under different RIP concentrations. Results indicated that RIP did not affect neutrophil viability and did not induce the release of MPO and LDH (Fig. 3). Therefore, neutrophils play a key role in the anti-infective effects of RIP and RIP may play a protective role in vivo by indirectly affecting neutrophils.

The pathogenicity of LAC is associated with toxin production. *S. aureus*, especially the CA-MRSA strain, can overcome neutrophil-mediated phagocytosis and succeed in evading destruction by neutrophils. Ultimately, neutrophil lysis ensues^29, 30^. Moreover, the *S. aureus* surviving inside neutrophils facilitates neutrophil lysis, which then results in the release of cell contents that promote local inflammation^29, 30^. Understanding the molecular mechanisms by which *S. aureus* avoids neutrophil-mediated responses and induces neutrophil lysis may provide insights into the effects of the *agr* inhibitor in vivo. The survival of *S. aureus* within neutrophils has been reported to undergo programmed necroptosis^17^. We postulated that RIPK1/RIPK3/MLKL-mediated necroptosis is likely involved in LAC-induced cytotoxicity. Our results confirmed that LAC or LAC supernatant can increase the level of pMLKL in human neutrophils, and the effect can be prevented by NSA or Nec (Fig. 4). However, HKLAC did not induce the phosphorylation of MLKL. Meanwhile, NSA or Nec also significantly reduced the supernatant-induced lysis of neutrophils dose-dependently. All the above-mentioned data indicated that the *S. aureus* culture supernatant may participate in the neutrophil necroptosis signaling pathway.

Studies have reported that PSMs are cytolytic for neutrophils in the micromolar range^11^. Through fluorescent-labeled *S. aureus*, Surewaard et al. found that PSMα contributes to neutrophil cell death and raises *S. aureus* survival^15^. Therefore, seven toxins of PSMs were synthesized to investigate whether these PSMs can induce neutrophil necroptosis. Our studies demonstrated that PSMα1, 2, and 3 can induce the MLKL phosphorylation and increase neutrophil cytotoxicity significantly (Fig. 5). TEM and cytotoxicity results also showed that NSA, as an MLKL inhibitor, can significantly reverse the neutrophil lysis caused by PSMα1. Moreover, culture supernatants harvested from the *agr* or *psmα* mutant decreased LDH release and the expression level of pMLKL, which further confirmed the effect of PSMα on inducing neutrophil necroptosis.

TNFα plays an important role in cell necrotic death by RIPK3 signaling pathway^31, 32^. TNFα also induces neutrophil necroptosis^27, 28^. To figure out whether or not TNFα participates in PSMα1-induced necroptosis in neutrophil cells, the cells were pretreated with anti-TNFα and subsequently stimulated with PSMα1. Our results showed that PSMα1-induced necroptosis was inhibited concentration-dependently with the increase in anti-TNFα as shown in Fig. 6. This finding definitely indicated that PSMα1 induced necroptosis depends on autocrine TNFα.

Until now, it’s still unclear how PSMα1 induces TNFα secretion in neutrophil. Cytolytic peptides PSMα peptides are more selective for FPR2 than for FPR1^33^. FPR2 is a specific receptor for PSMα^34, 35^, although most bacteria-derived formyl peptides are more potent at FPR1 than FPR2. Moreover, the virulence regulator *agr* controls the staphylococcal capacity to activate human neutrophils via the FPR2^36^. Basing on these reports, we speculated that PSMα1 may induce TNFα secretion by FPR2 in neutrophils. To verify this hypothesis, we pretreated with FPR2 inhibitor WRW4 and then detected the necroptosis of neutrophils induced by PSMα1. Our data showed that blocking FPR2 by WRW4 also markedly prevented PSMα1-induced neutrophil necroptosis and decreased the level of TNFα in cell culture (Fig. 6) in a concentration dependent manner. All these results indicated that PSMα1 induced TNFα secretion through FPR2 and autocrine TNFα subsequently caused neutrophil necroptosis.

Interfering with the bacterial virulence release pathways is a potential approach because of the ability of this strategy to bypass evolutionary pressure on the bacterium to develop resistance. This ability is absent in traditional bactericidal strategies^8, 9, 37^. Our data firstly indicated *S. aureus-*secreted PSMα can induce neutrophil necroptosis by promoting TNFα autocrine and exacerbate severe tissue damage. Moreover, blocking *agrA* and *psmα* expression significantly suppressed the neutrophil necroptosis induced by *S. aureus* and effectively prevented pneumonia through the *psmα* mutant strain, with RIP as *agr* inhibitor and Nec as RIPK1 inhibitor. Hence, the blockage of the *agr* system is a promising strategy for inhibiting the neutrophil necroptosis of infected mice and improving the clearance of *S. aureus* from infected mice.

## Materials and methods

### Bacteria and agents

*S. aureus* ATCC29213 and *S. epidermidis* ATCC14990 were obtained from the Chinese National Center for Surveillance of Antimicrobial Resistance. Mu50 ATCC700699 was purchased from MicroBiologics (Minnesota, USA). LAC (USA 300) and its mutant strains (*Δpsmα* and *Δagr*) were generous gifts from Michael Otto (National Institute of Allergy and Infectious Diseases, MD). MRSA XJ75302 and *methicillin-resistant S. epidermidis* (MRSE) XJ75284 were obtained from the clinical laboratory of Xijing Hospital (Xi’an, China). The *S. aureus* strains used are listed in Supplementary Table 2.

Vancomycin, oxacillin, ciprofloxacin, ceftazidine, and levofloxacin were purchased from the National Institute for the Control of Pharmaceutical and Biological Products (Beijing, China). RIP (CH_3_CO-YKPVTNF-CONH_2_) and PSMs (Supplementary Table 1) were synthesized and the purity of the peptide is >95%. NSA (Calbiochem, USA) is a pharmacological inhibitor of MLKL. Nec (Calbiochem, USA) is an inhibitor of RIPK1. WRW4 (Alomone Labs, USA) is an antagonist of FPR2.

### Antimicrobial susceptibility and growth assay

Minimal inhibitory concentrations (MICs) of peptides and antibiotic were performed in sterile, flat-bottomed 96-well microplates according to the broth microdilution guidelines of Clinical and Laboratory Standards Institute (CLSI)^38^.

To determine the growth curve for bacteria, the working suspension of the inoculum (2 × 10^7^ CFU/mL, 160 μL) was cultivated in the automated Bioscreen C system (Lab systems Helsinki, Finland), using a MH broth culture medium. And 40 μL synthetic peptide solution was added to strain cultures to a final concentration of 250, 500, or 1000 μg/mL. The optical density of the cell suspensions was measured at 630 nm in regular intervals of 1 h for 22 h.

### Pneumonia model and BALF collection

BABL/c mice weighing ~20 g were housed for 7 days prior to inoculation. Mice were anesthetized intraperitoneal injection with 400 mg/kg chloral hydrate and hung in an upright position, and inoculated with LAC (3 × 10^7^ CFU) in a volume of 30 μL PBS into the right nares. Keep the infectious mice in the upright position for 1 min. Control mice received 30 μL sterile PBS. Treatment was initiated at 1 and 6 h after infection. To observe the weight recovery, the mice were weighed at 0, 1, 2, 3, 5, and 7 days (at a fixed time) after infection. Mice were monitored every 6 h after infection, and survivors were euthanized for 7 days. Lungs were harvested at 24 h after infection. The right lung was homogenized in 1 mL of PBS, and serial dilutions were plated in duplicate on agar plates for evaluating bacterial burden in the infected organs. The left lung of infected mice were inflated with 10% buffered formalin, processed, stained with HE stain, and analyzed microscopically. All HE-stained sections were scored by a pathologist who was blinded to study-group attribution. The total pathologic score for each mouse was calculated as the sum of scores from each category for that individual.

Pneumonia mice were anesthetized with an intraperitoneal injection of chloral hydrate (400 mg/kg). The trachea was cannulated (22 GA Insyte, Becton Dickinson), and 1 mL cold PBS was infused intratracheally and withdrawn. This procedure was repeated three times, resulting in a total volume of 2.5 mL. The bacterial CFU in BALF were quantified by plating serial dilutions on MH agar plates and enumerating colonies. To quantify the degree of pulmonary edema, we detected the lung water content as described previously. The lung weight was measured immediately after its excision (wet weight). The lung tissue was then dried in an oven at 60 °C for 72 h. Lung wet/dry weight (W/D) ratio = wet weight/dry weight.

### Quantitative RT-PCR

The in vivo *agrA* and *psmα* expression levels were measured by directly extracting the MRSA RNA from the lung BALF of infected BALB/c mice^39^. Total RNA was isolated with Trizol and reverse transcription was performed with reverse transcriptase according to the manufacturer’s instructions. RT-PCR was performed using multiple kits (SYBR Premix Ex TAQ, Takara Bio, Japan) according to the manufacturer’s instructions. To determine the relative expression level of mRNA, each gene was normalized to the expression level of the housekeeping gene *16SrRNA*. The primers used in the present study are listed in Supplementary Table 3.

### Macrophage or neutrophil depletion in mice

Macrophages were depleted by a single tail vein administering 150 μL clodronate liposomes (Clodronate Liposomes. org, Netherlands). Then mice were challenged with LAC (3 × 10^7^ CFU) by intraperitoneal injection. Infected-mice were sacrificed at 0, 24, 48, 96, and 120 h later. Macrophage depletion was confirmed by flow cytometry in peritoneal lavage fluid.

Mice were rendered neutropenic by a single tail vein injection cyclophosphamide (CTX, 200 mg/kg). Peritoneal lavage fluid was collected and the percentage of macrophage was detected by flow cytometry after the mice were intraperitoneal administered with LAC (3 × 10^7^ CFU). Blood was collected from the retro-orbital sinuses of anesthetized to determine the extent of neutropenia at 0, 24, 48, 96, and 120 h after injection. The blood was analyzed with an Abbott Cell-Dyn 3700 system.

### Neutrophil isolation and co-culture with *S. aureus*, supernatant, or PSM

Human neutrophils were isolated from the blood of healthy volunteers by standard Ficoll/Histopaque gradient centrifugation. The resulting preparation was found to be over 98% neutrophils according to Wright-Giemsa staining. Trypan blue staining to identify cell viability was over 95%. Neutrophil cells were grown in RPMI Medium 1640 (Gibco) with 10% fetal bovine serum. Human neutrophils were challenged with LAC at multiplicities of infection (MOI 10:1; CFU of *S. aureus*: neutrophils). Neutrophil cells were pretreated 1 h with NSA (1, 3 or 10 μM) or Nec (50, 100 or 200 μM) and then were stimulated with LAC (WT, *Δpsmα*, *Δagr*) supernatant or Heat-killed LAC (HKLAC) for 1 h at 37 °C and 5% CO_2_.

### MPO activity

Neutrophils (1 × 10^6^) was treated with 10, 30, 100, 300, or 1000 μg/mL RIP or 50 ng/mL LPS at 37 °C in 5% CO_2_ for 30 min. Cells were collected by centrifugation at 1000 rpm for 5 min and were lysed to getting cell-free supernatant for MPO activity. MPO activity of neutrophil was measured by spectrophotometer according to the instructions of MPO assay kit.

### Human TNFα enzyme-linked immunosorbent assay

Neutrophil cells were stimulated with 1 μM PSMα1 at 37 °C for 1 h after pretreated with 1.25, 2.5, 5, or 10 μΜ WRW4 for 1 h. Then the level of human TNFα in the cell culture supernatant was assessed using an enzyme-linked immunosorbent (ELISA) kit (Boster Biological Technology, China) after centrifugation (1000 rpm, 10 min).

### Cytotoxicity detection

Neutrophils was treated with 10, 30, 100, 300, or 1000 μg/mL RIP at 37 °C in 5% CO_2_. Cell viability was assessed 24 h later using Cell counting Kit 8 (CCK-8, Dojingdo Laboratories, Japan) according to the manufacturer’s protocol. The cell-free supernatant is used to detect the release of lactate dehydrogenase (LDH) according to the manufacturer’s protocol (Cytotoxicity Detection Kit, Roche).

Neutrophil cells were respectively stimulated with PSM peptide for 1 h at 37 °C and then centrifugated (1000 rpm, 10 min) after pretreated with or without NSA, Nec, WRW4, anti-TNFα for 1 h. The neutrophil cell culture supernatant is used to detect the release of LDH.

### Flow cytometry analysis

Mice were sacrificed by cervical dislocation. The peritoneal cavity was lavaged with 3 mL of ice-cold sterile PBS. The buffer containing resident peritoneal cells was slowly withdrawn. Cells collected by concentration were suspended in fluorescence-activated cell sorter buffer (10% fetal bovine serum and 0.1% sodium azide in PBS) and stained for 30 min at 4 °C. The macrophages were stained with the F4/80-FITC macrophage specific glycoprotein. The percentage of F4/80-positive cells was determined by counting cell numbers.

Lung sections were incubated in 2.5 mg/mL collagenase with 0.5 mg/mL DNase-1 (Sigma-Aldrich) at 37 °C for 60 min and red blood cells were lysed with ammonium chloride potassium containing lysing buffer (Gibco) to aquire single-cell suspensions. Cells were suspended in fluorescence-activated cell sorter buffer and stained for 30 min at 4 °C. Combinations of fluorescein isothiocyanate-labelled (FITC) anti-Ly-6G (Gr-1; RB6-8C5; Biolegend) and propidium iodide (PI) were used. Macrophages (F4/80^+^) and neutrophils (neutrophils; Ly6G^+^) were enumerated using FlowJo V10.

### Western blot analysis

*S. aureus* supernatant was obtained by centrifugation and filtration. HKLAC was obtained by incubating LAC cells in PBS (1 × 10^9^ CFU/mL) at 65 °C for 2 h to inactivate the bacteria. Neutrophil lysates were run on bolt 4–12% Bis-Tris Plus gels (Life Technologies) and transferred to polyvinylidene difluoride membranes (Millipore). Then the membrane was blocked with 5% milk in TBST (Tris-buffered saline plus Tween) for 1.5 h at room temperature. Immunodetection was performed using anti-phospho-MLKL (Ser358) (Abcam, Cambridge, UK), anti-MLKL (Abcam, Cambridge, UK), anti-TNFα (R&D Systems, USA), and β-actin (Sigma Aldrich Chemical Co, USA) antibodies followed by secondary antibodies conjugated to horseradish peroxidase (Santa Cruz Biotechnology Inc, USA).

### Transmission electron microscopy

The cells were fixed with ice-cold 2.5% glutaraldehyde in PBS (pH 7.3) at 4 °C for 4 h. Fixed cells were post-fixed in 2% OsO_4_, dehydrated in graded alcohol, embedded in Epon 812 (Electron Microscopy Sciences, Fort Washington, PA, USA), sectioned with ultramicrotome, and stained with uranyl acetate and lead citrate. Images were acquired under a laser confocal microscope (FV1000, Olympus).

### Statistical analysis

Results are expressed as mean (Figs. 1c, d, f and 7b), mean ± standard deviations (Figs. 1e and 3b, d), or mean ± standard error of the mean (Figs. 1a, 2, 3f–i, 4c, e, 5b, d, 6a, b, e and 7d). Two-way analysis of variance (ANOVA) was used for the data presented in Figs. 1a, 2c, d, 3b, d, f, and 5b, d, e. Comparisons of three or more groups were performed using one-way ANOVA (Figs. 1c–f, 2a, b, 3g–i, 4c, e, 5f, 6a, b, e and 7b, d). Survival curves were calculated by the Kaplan–Meier method (Figs. 1b, 3a, c, e and 7a). Statistical analyses were done using Prism software (version 6; GraphPad, CA). A probability value of *P* < 0.05 was considered statistically significant.

## Ethics statement

Animal experiments were carried out in strict accordance with National Institutes of Health guidelines for the care and use of laboratory animals and approved by the Institutional Animal Care and Use Committee of the Fourth Military Medical University. The present study was approved by the Medical Ethical Committees of the Fourth Military Medical University (XJYYLL-2014489).

## Electronic supplementary material

Supplementary Tables and Figure
