# Supplementary material for: Inhibiting PSMα-induced neutrophil necroptosis protects mice with MRSA pneumonia by blocking the agr system
Source: Cell Death Dis. 2018 Mar 2;9(3):362. doi: 10.1038/s41419-018-0398-z (PMC5834619; doi:10.1038/s41419-018-0398-z)
Supplement: Supplementary file 1 — Supplementary Tables and Figure [file 41419_2018_398_MOESM1_ESM.docx]

Supplementary Tables & Figure

Supplementary Table 1 PSMs used in this study.

| **Peptide** | **Sequence** | **MW** | **Purity (%)** |
| --- | --- | --- | --- |
| PSMα1 | fMGIIAGIIKVIKSLIEQFTGK | 2287.85 | 95.17 |
| PSMα2 | fMGIIAGIIKFIKGLIEKFTGK | 2305.92 | 95.16 |
| PSMα3 | fMEFVAKLFKFFKDLLGKFLGNN | 2635.19 | 95.95 |
| PSMα4 | fMAIVGTIIKIIKAIIDIFAK | 2199.83 | 95.17 |
| PSMβ1 | fMEGLFNAIKDTVTAAINNDGAKLGTSIVSIVENGVGLLGKLFGF | 4524.25 | 95.91 |
| PSMβ2 | fMTGLAEAIANTVQAAQQHDSVKLGTSIVDIVANGVGLLGKLFGF | 4484.19 | 96.88 |
| δ-toxin | fMAQDIISTISDLVKWIIDTVNKFTKK | 3036.61 | 96.02 |

Supplementary Table 2 *Staphylococcus* *aureus* strains used in the study.

| ***S*trains** | **Description** | **Source** |
| --- | --- | --- |
| *S. aureus* ATCC 29213 | Oxacillin-susceptible strain | ATCC |
| LAC (USA300) | Typical community-acquired strain of MRSA | Michael Otto (NIH) |
| LAC (*∆agr*) | USA300 with accessory gene regulator (agr) mutant | Michael Otto (NIH) |
| LAC (*∆psmα*) | USA300 with phenol-soluble modulinα mutant | Michael Otto (NIH) |
| LAC (*∆psmβ*) | USA300 with phenol-soluble modulinβ mutant | Michael Otto (NIH) |
| Mu50 ATCC700699 | MRSA strain with Vancomycin-intermediate resistance | ATCC |
| MRSA XJ75302 | Hospital-acquired strain | Xijing Hospital |

Supplementary Table 3 Primers used in this study.

| Gene | Forward sequence(5’-3’) | Reverse sequence(5’-3’) |
| --- | --- | --- |
| *16S rRNA* | CTTTATGGGATTTGCTTGA | GTCGTGAGATGTTGGGTTA |
| *psmα* | TATCAAAAGCTTAATCGAACAATTC | CCCCTTCAAATAAGATGTTCATATC |
| *psmβ* | CTAGCAGAAGCAATCGCAAA | AACCCACACCGTTAGCAACG |
| *agrA* | TGATAATCCTTATGAGGTGCTT | CACTGTGACTCGTAACGAAAA |





Supplementary Figure S1. Growth curves of RIP and vancomycin for six strains. 40 μL synthetic peptide solution was added to strain cultures (2×10^7^ CFU/mL, 160 μL) to a final concentration of 250, 500 or 1000 μg/mL. The optical density of the cell suspensions was measured at 630 nm in regular intervals of 1 h for 22 h.
